# Recombinant Vaccinia Virus Expressing *Plasmodium berghei* Apical Membrane Antigen 1 or Microneme Protein Enhances Protection against *P. berghei* Infection in Mice

**DOI:** 10.3390/tropicalmed7110350

**Published:** 2022-11-04

**Authors:** Min-Ju Kim, Ki-Back Chu, Su-Hwa Lee, Hae-Ji Kang, Keon-Woong Yoon, Md Atique Ahmed, Fu-Shi Quan

**Affiliations:** 1 Department of Biomedical Science, Graduate School, Kyung Hee University, Seoul 02447, Korea; 2 Medical Research Center for Bioreaction to Reactive Oxygen Species and Biomedical Science Institute, School of Medicine, Graduate School, Kyung Hee University, Seoul 02447, Korea; 3 Department of Medical Zoology, School of Medicine, Kyung Hee University, Seoul 02447, Korea; 4 ICMR-Regional Medical Research Centre, NE Region, Dibrugarh 786010, Assam, India

**Keywords:** *Plasmodium berghei*, recombinant vaccinia virus (rVV), vaccine, apical membrane antigen 1, microneme protein

## Abstract

Recombinant vaccinia viruses (rVV) are effective antigen delivery vectors and are researched widely as vaccine platforms against numerous diseases. Apical membrane antigen 1 (AMA1) is one of the candidate antigens for malaria vaccines but rising concerns regarding its genetic diversity and polymorphism have necessitated the need to search for an alternative antigen. Here, we compare the efficacies of the rVV vaccines expressing either AMA1 or microneme protein (MIC) of *Plasmodium berghei* in mice. Mice (BALB/c) were immunized with either rVV-AMA1 or rVV-MIC and subsequently challenge-infected with *P. berghei.* Compared to the control group, both antigens elicited elevated levels of parasite-specific antibody responses. Immunization with either one of the two vaccines induced high levels of T cells and germinal center B cell responses. Interestingly, rVV-MIC immunization elicited higher levels of cellular immune response compared to rVV-AMA1 immunization, and significantly reduced pro-inflammatory cytokine productions were observed from the former vaccine. While differences in parasitemia and bodyweight changes were negligible between rVV-AMA1 and rVV-MIC immunization groups, prolonged survival was observed for the latter of the two. Based on these results, our findings suggest that the rVV expressing the *P. berghei* MIC could be a vaccine-candidate antigen.

## 1. Introduction

Malaria is one of the diseases that account for a large proportion of disease-related mortality worldwide, particularly in Africa. Statistically, on a global scale, approximately 241 million malaria cases and 627,000 malaria-related deaths were reported in 2020 [1]. However, despite decades of research, commercialized vaccines are unavailable. RTS,S/AS01 vaccine based on the *P. falciparum* circumsporozoite protein (CSP) is the most advanced malaria vaccine reported to date, but its recent phase 3 clinical trial results were disappointing. Specifically, the vaccines were initially protective, but their efficacies drastically waned over 7 years of follow-up [2]. Given this circumstance, additional efforts using different methods of approach are required. One such approach is the use of modified vaccinia virus Ankara (MVA) vectors.

Historically, vaccinia viruses were used as smallpox vaccines in the 20th century and were integral to eradicating smallpox [3]. The MVA is a replication-deficient attenuated form of the vaccinia virus that is capable of expressing recombinant genes, thus highlighting their use as potent viral vector vaccines. The MVA vaccine platform is highly regarded as being safe and immunogenic, with the latter of the two aspects being subjected to change depending on the immunization dose and immunization regimen [4]. As such, a large number of studies involving MVA vaccines were conducted in an attempt to develop an efficacious malaria vaccine, and positive findings were confirmed through numerous clinical trials. A prime-boost regimen using the recombinant chimpanzee adenovirus 63 (ChAd63) and MVA vaccines encoding the *P. falciparum* CSP were confirmed to be safe and immunogenic in a clinical trial [5]. Using the same approach, ChAD63-MVA vaccines encoding the apical membrane antigen 1 (AMA1) induced potent T cell and antibody responses in vaccinees [6]. Another clinical study reported that heterologous immunization with ChAD63-MVA vectors expressing either the merozoite surface protein 1 (MSP1) or AMA1 elicited an increase in antigen-specific seroreactivity in vaccinees, even after controlled human malaria infection [7]. Despite these promising findings, many of the surface proteins of *Plasmodium* spp. are highly polymorphic and often result in vaccine-induced immune responses being allele-specific. This genetic diversity, in turn, compromises the vaccine efficacy by exerting selection pressure towards escape mutation [8]., Other antigen candidates must be explored to address these issues.

Microneme (MIC) is a secretory organelle commonly found in Apicomplexan parasites, and several studies have reported the immunogenicity of these micronemal antigens. Antibodies raised against the *Eimeria tenella* microneme protein 2 (MIC2) inhibited the parasitic invasion of host cells, and immunizing chickens with the MIC2-expressing DNA vaccines lessened the severity of infection [9]. Similarly, antibodies raised against the micronemal protein 1 of another Apicomplexan parasite, *Babesia bigemina* neutralized merozoite invasion when tested in vitro [10]. Chimeric antigen prepared by fusing the microneme and rhoptry proteins of *Neospora caninum* significantly reduced the cerebral parasite burden and protected mice against neosporosis [11]. In the case of *Plasmodium* spp., only a handful of malaria vaccine studies have evaluated the immunogenicity of micronemal antigens. Antibodies raised against the *P. falciparum* glycosylphosphatidylinositol-anchored micronemal antigen (GAMA) were capable of inhibiting parasitic invasion [12]. Similarly, the *P. vivax* GAMA protein vaccine induced both cellular and humoral immune responses in individuals naturally infected with *P. vivax*, thus highlighting their further development as blood-stage vaccine candidates [13]. To date, a *P. berghei* microneme antigen-based vaccine study has yet to be conducted. Furthermore, the rodent malaria *P. berghei* shares multiple features resembling those of the human malaria *P. falciparum* [14], making them highly desirable for vaccine studies. Here, we evaluate the efficacy of recombinant vaccinia virus vaccines expressing the micronemal antigen. We also compare the efficacy of this vaccine to that of a vaccinia-based vaccine expressing the AMA1 antigen reported in our previous study [15], which conferred suboptimal protection.

## 2. Materials and Methods

### 2.1. Animals, Parasites, Cells, and Antibodies

To maintain the *Plasmodium berghei* ANKA strain and immunization vaccines, female seven-week-old BALB/c mice were purchased from NARA Biotech (Seoul, Korea). Mice were housed in an institute-approved facility with a 12 h day and night cycle, with easy access to food and water. Nesting material and enrichment were also provided to prevent the development of abnormal behaviors.The *P. berghei* ANKA strain was used for challenge infection and antigen preparation. CV1 cells (CCL-70) and Vero cells (CCL-81) were purchased from the American Type Culture Collection (Manassas, VA, USA). Both cell lines were cultured using Dulbecco’s Modified Eagle Media, supplemented with fetal bovine serum and penicillin/streptomycin at 37 °C, 5% CO_2_ for production and characterization of rVVs. Sera of *P. berghei*-infected mice polyclonal antibodies were collected through retro-orbital plexus puncture. Horseradish peroxidase (HRP)-conjugated goat anti-mouse immunoglobulin G (IgG) was purchased from Southern Biotech (Birmingham, AL, USA).

### 2.2. P. berghei Antigen Preparation

The *P. berghei* ANKA strain antigen was prepared as described previously [16]. Briefly, red blood cells (RBCs) were collected from the whole blood of *P. berghei*-infected mice with parasitemia exceeding 20% by low-speed centrifugation. Pelleted RBCs were lysed with an equal volume of 0.15% saponin in PBS, and the released parasites were pelleted and washed 3 times with PBS. Parasites were sonicated at 30 s, at 40% amplitude, for two cycles on ice and stored at −20 °C until used. The *P. berghei* antigen was used as coating antigens for enzyme-linked immunosorbent assay (ELISA) and as the stimulant for flow cytometric analysis.

### 2.3. Generation of Recombinant Vaccinia Virus

To generate the *P. berghei* recombinant vaccinia virus, codon-optimized *P. berghei* AMA1 or MIC genes containing *EcoR*I and *Hind*III restriction enzyme sites were purchased from GenScript (Piscataway, NJ, USA). The codon-optimized AMA1 and MIC genes were cloned into the green fluorescent protein (GFP)-expressing pRB21 vaccinia virus vectors, which were kindly provided by Dr. Sang-Moo Kang at Georgia State University (Atlanta, GA, USA). CV-1 cells were cultured in DMEM supplemented with 10% inactivated FBS and 1% penicillin/streptomycin antibiotics. Confluent monolayers of CV-1 cells initially infected vRB12 using DMEM media without antibiotics for 1 h at 37 °C. Afterward, cells were transfected with pRB21-AMA1, pRB21-MIC, or GFP-expressing pRB21 vector control using the Lipofectamine LTX/PLUS™ (Thermo Fisher, Waltham, MA, USA) transfection reagent and incubated for 2–3 days at 37 °C. Cells were monitored under a fluorescent microscope to evaluate successful transfection. After 3 days, recombinant vaccinia virus was harvested and centrifuged at 2000 rpm for 10 min. Cell culture supernatants were discarded, and pelleted cells were resuspended in DMEM media, and stored at −80 °C. Frozen cells were repeatedly thawed in a 37 °C water bath and re-frozen at −80 °C. Recombinant vaccinia viruses were released from CV-1 cells via sonication and stored at −80 °C until use. To confirm the presence of AMA1 and MIC in the recombinant vaccinia virus, Western blots were performed, and membranes were sequentially probed with a primary antibody (anti-*P. berghei* polyclonal antibody) and then a secondary antibody (HRP-conjugated anti-mouse IgG). As a loading control for Western blots, membranes were probed with monoclonal beta-actin antibody (SC-47778; Santa Cruz Biotechnology, Dallas, TX, USA). Bands were developed using the enhanced chemiluminescence (ECL) purchased from Bio-Rad (Bio-Rad #1705061; Hercules, CA, USA) and visualized using ChemiDoc (Bio-Rad, Hercules, CA, USA). 

### 2.4. Recombinant Vaccinia Virus Plaque Assay

Recombinant vaccinia virus titers were determined using Vero cells. Confluent monolayers of Vero cells cultured in 12-well plates were infected with the vaccinia virus diluted in serum-free DMEM media for 1 h at 37 °C. Overlay media was made with DMEM media containing 2% FBS and 1% agarose. After 1 h, the virus inoculum in each well was aspirated, and 1 mL of overlay media was added to each well. Plates were incubated at 37 °C, 5% CO_2,_ until noticeable plaque development occurred, which enables quantification.

### 2.5. Immunization and Challenge

Seven-week-old female BALB/c mice were randomly divided into four experimental groups (naïve, naïve challenge, AMA1 rVV, MIC rVV; *n* = 8 per group) to evaluate the protective efficacies of PbAMA1 and PbMIC rVV vaccines. Mice were intramuscularly immunized with AMA1 (Prime: 1.8 × 10^7^, 1st boost: 6.8 × 10^7^, 2nd boost: 1.3 × 10^8^ PFU/mouse) or MIC (Prime: 1.8 × 10^7^, 1st boost: 6 × 10^7^, 2nd boost: 1 × 10^8^ PFU/mouse) rVV at weeks 0, 4, and 8. Four weeks after the 2nd boost immunization, mice were challenged with 0.5%/100 uL in PBS (0.5 × 10^4^) of *P. berghei* by intraperitoneal (IP) injection, as described previously [15]. Four mice from each group were euthanized at 6 days post-infection (dpi) for blood, spleen, and inguinal lymph node sample collection. The remaining four mice were observed daily to monitor changes in body weight and parasitemia in blood. The human intervention point was set as 20% of initial body weight loss, and mice that reached this point were humanely euthanized using CO_2_.

### 2.6. Antibody Responses in Sera

Mice sera were collected from all groups 4 weeks after prime, 1st boost immunization, and 2nd boost immunization using a retro-orbital plexus puncture. Sera from naïve mice were used as a negative control. Antibody responses against *P. berghei* antigen, AMA1 rVV, and MIC rVV were determined by enzyme-linked immunosorbent assay (ELISA), as described previously [17]. Briefly, 96-well immunoplates were coated with 100 μL of *P. berghei* antigen, AMA1 rVV, or MIC rVV at a final concentration of 2, 0.5, and 0.5 μg/mL, each respectively in 0.05 M, pH 9.6 carbonate bicarbonate buffer per well at 4 °C overnight. Afterwards, 100 μL of serially diluted serum samples were added into the respective wells and incubated at 37 °C for 1.5 h. HRP-conjugated goat anti-mouse IgG (100 μL/well, diluted 1:2000 in PBS) was used to determine the *P. berghei*-specific IgG antibody response. O-phenylenediamine was dissolved in citrate substrate buffer, and OD_492_ was measured using an EZ Read 400 microplate reader (Biochrom Ltd., Cambridge, UK).

### 2.7. Immune Cell Responses by Flow Cytometry

To determine the immune cell responses, the levels of T and B cell populations from the blood and inguinal lymph nodes (ILN) of mice were evaluated by flow cytometry. The ILNs of mice were aseptically removed, and lymphocytes were acquired as previously described [18], and placed on top of a cell strainer. The cell strainer containing the ILNs was placed on a Petri plate and immersed in 1 mL PBS. Lymph nodes were thoroughly homogenized using the plunger portion of a syringe, and the resulting homogenates were carefully collected. CD4^+^ and CD8^+^ T cells from the blood were detected at 6 dpi. The levels of CD8^+^ T cells and germinal center (GC) B cell proliferation were measured from ILN cells at 6 dpi. Immune cell populations (1 × 10^6^ cells per sample) were resuspended in PBS and stimulated with 0.05 μg of *P. berghei* antigen for 2 h at 37 °C before staining with surface antigen antibodies. Cells were incubated in FACS staining buffer (2% bovine serum albumin and 0.1% sodium azide in 0.1 M PBS) for 15 min at 4 °C with Fc Block antibody (clone 2.4G2; BD Biosciences, San Jose, CA, USA). Afterwards, cells were incubated with the fluorophore-conjugated cell surface antigen antibodies (CD3e-PE-Cy5, CD4-FITC, CD8a-PE, B220-FITC, GL7-PE; BD Biosciences, CA, USA) at 4 °C for 30 min. Stained cells were acquired using a BD Accuri C6 Flow Cytometer (BD Biosciences, CA, USA), and the data were analyzed using C6 Analysis software (BD Biosciences, CA, USA).

### 2.8. Inflammatory Cytokine Production in the Spleen

At 6 dpi, the mice were euthanized, and spleens were harvested. Single cell suspensions of splenocytes were prepared from individual spleens by homogenization. Briefly, spleens were firmly pressed using two frosted glass slides in RPMI media. Homogenates were carefully collected into 15 mL conical tubes and centrifuged at 2000 rpm for 5 min. The resulting supernatants were collected to detect inflammatory cytokines interferon-gamma (IFN-γ) and tumor necrosis factor-alpha (TNF-α). The levels of splenic cytokine expressions were detected using the BD OptEIA IFN-γ, TNF-α ELISA kits (BD Biosciences, San Jose, CA, USA). Cytokine concentrations were quantified following the manufacturer’s instructions.

### 2.9. Parasitemia

Infected mouse blood samples were collected via retro-orbital plexus puncture. After resuspending 2 μL of the blood samples into microcentrifuge tubes containing 500 U/mL of heparin premixed in 100 μL of PBS, RBCs were stained using 1 μL SYBR Green Ι (Invitrogen, Carlsbad, CA, USA). Samples were incubated at 37 °C for 30 min in the dark. After incubation, 0.1 M PBS was added to each sample and flow cytometry was performed [19].

### 2.10. Statistics

All parameters were recorded for individuals in all the groups. The data were presented as mean ± SD, and statistical significances between groups. They were analyzed by one-way analysis of variance (ANOVA) and Student’s *t*-test using GraphPad Prism version 6.0 (GraphPad Software, San Diego, CA, USA). *p* values (* *p* < 0.05) were considered statistically significant.

## 3. Results

### 3.1. Gene Cloning and Recombinant Vaccinia Virus Generation

**Figure 1:**
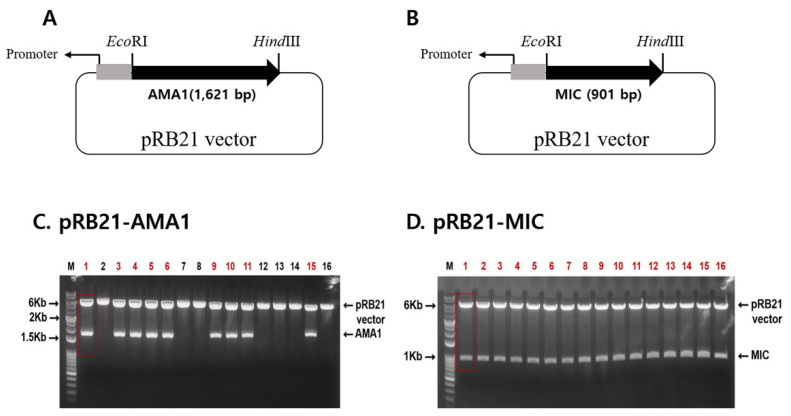
Gene cloning. To generate *P. berghei* AMA1 and MIC recombinant vaccinia virus (rVV), gene cassettes with promoters and recombination sites were designed, shown as a schematic diagram (**A**,**B**). PbAMA1 or PbMIC genes were cloned into the pRB21 vector, and clones pRB21-AMA1 (**C**) and pRB21-MIC (**D**) were confirmed by restrictive enzyme digestion with *EcoR*Ⅰ and *Hind*Ⅲ. The red color font denotes successful clones.

**Figure 2:**
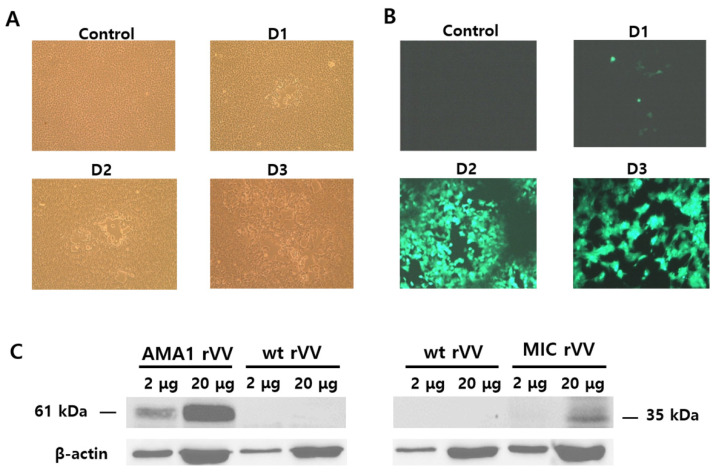
Transfection of CV-1 and confirming the production of rVV. The pRB21-PbAMA1 and pRB21-PbMIC DNA constructs were transfected into CV-1 cells, along with the vaccinia virus, to generate rVV-AMA1 and rVV-MIC. Representative images of rVV transfection under brightfield (**A**) and fluorescent microscope (**B**) are illustrated over the course of 3 days. AMA1 and MIC rVVs were identified by a Western blot using a *P. berghei* polyclonal antibody and a wild-type vaccinia virus (wt VV) control (**C**).

**Figure A1:**
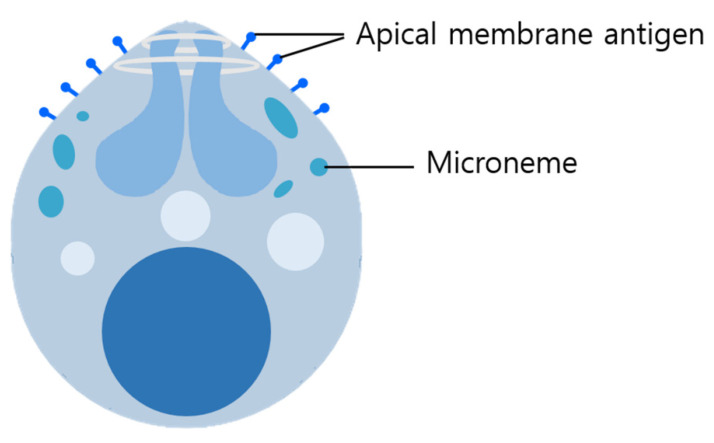
The diagram of the *Plasmodium* organism. The diagram describes the Plasmodium organism and the specified target gene (AMA1, MIC).

An illustration depicting the location of the AMA1 and MIC antigens within the *Plasmodium* parasites was provided (Figure A1). To generate recombinant vaccinia viruses expressing the *P. berghei* AMA1 or MIC, the AMA1 or MIC in the pRB21 vector were designed (Figure 1A,B). AMA1 (1621 bp) or MIC (901 bp) DNAs were PCR amplified and cloned into a pRB21 vector. Successful clones were confirmed by restriction enzyme digestion (Figure 1C,D). Clones PbAMA1 and PbMIC DNAs were transfected into CV-1 cells for rVV-AMA1 and rVV-MIC production. Representative brightfield and fluorescence images for transfection were provided. Cellular lysis, identified by the empty regions in the cell monolayer, was not observed in the non-transfected control. Lysed cells were detectable at 1 dpi onward and a noticeable increase in cellular lysis was observed as time progressed (Figure 2A). Cells were also monitored daily under a fluorescent microscope, to confirm successful transfection of rVV-AMA1 and rVV-MIC. As expected, on day 0, non-transfected cells did not emit fluorescence. Low levels of green fluorescence emission were observed from cells at 1 dpi, thereby indicating successful transfection. By 2 dpi, a drastic increase in transfected cell populations was observed. The irregularly shaped dark regions indicate areas of cellular lysis. At 3 dpi, a substantial increase in cellular lysis was observable, consistent with the brightfield images (Figure 2B). A western blot was performed to confirm the presence of AMA1 and MIC in the recombinant vaccinia virus. Polyclonal *P. berghei* antibody was used to determine AMA1 and MIC expressions at molecular weights of 61 and 35 kDa, respectively (Figure 2C).

### 3.2. IgG Antibody Responses

**Figure 3:**
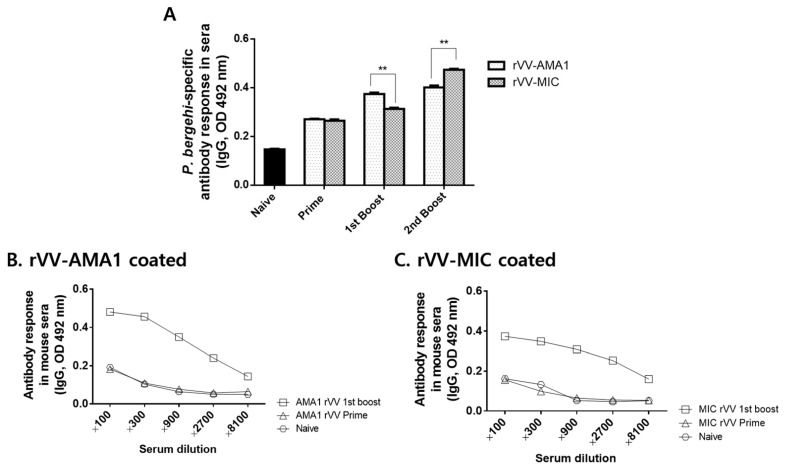
IgG antibody response in sera. On week 4 after prime, 1st boost, and 2nd boost immunization, the sera of mice were collected to investigate the levels of IgG antibody response. The 96-well immunoplates coated with *P. berghei* antigen (**A**), rVV-AMA1 (**B**), or rVV-MIC (**C**) were used to verify IgG antibody response by ELISA. Data are expressed as mean ± SD (** *p* < 0.01).

**Figure A2:**
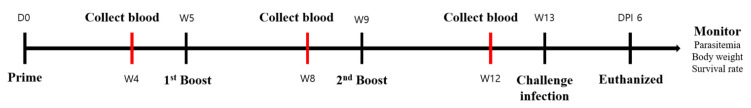
A schematic diagram depicting animal experimental schedule. Immunization intervals, blood collection, challenge infection, and euthanasia time points are indicated.

A schematic diagram depicting immunization and challenge infection schedules was provided (Figure A2). Sera were regularly collected from mice 4 weeks after prime, 1st boost, and 2nd boost immunizations to assess parasite-specific IgG antibody responses against *P. berghei* antigen. Differences in antibody responses elicited by the two vaccines after prime immunizations were negligible. At 4 weeks after the 1st boost immunization, antibody responses were slightly enhanced, with significantly greater levels induced by rVV-AMA1. However, after the 2nd boost immunization, the trend was reversed. Immunization with the rVV-MIC elicited a stronger antibody response reacting to the malaria antigen than rVV-AMA1 (Figure 3A). To determine the levels of IgG antibody responses that are reactive to the recombinant vaccinia virus, sera were collected at 4 weeks after the 1st boost immunization, and ELISA was performed (Figure 3B,C). As seen in Figure 3B,C, higher levels of IgG antibody responses were observed using rVV-AMA1 (Figure 3B) or rVV-MIC as coating antigens (Figure 3C) compared to naïve control.

### 3.3. CD4^+^, CD8^+^ T Cells and Germinal Center B Cell Response in the Blood and ILN

**Figure 4:**
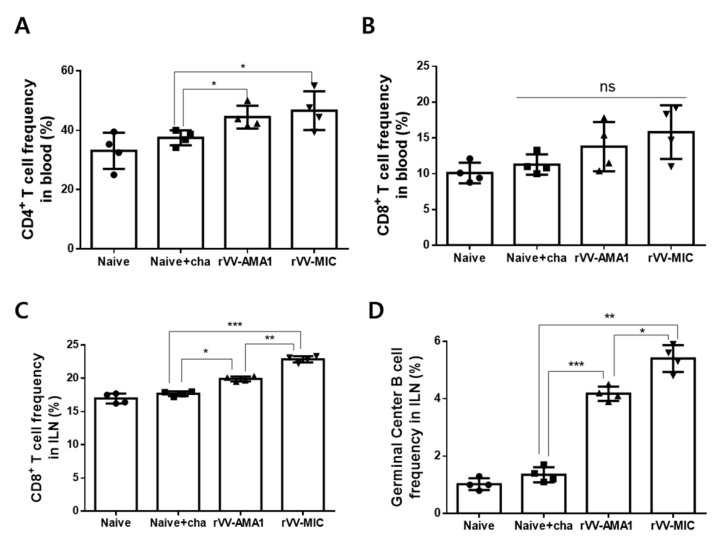
T cell and germinal center B cell response in blood and inguinal lymph nodes (ILN). Immunized mice (*n* = 8) were challenge-infected with *P. berghei,* and blood samples were collected at 6 dpi. Inguinal lymph nodes (ILN) from mice were collected at 6 dpi. After surface marker staining with the fluorophore-conjugated antibodies, CD4^+^ (**A**) and CD8^+^ (**B**) T cell responses in the blood and CD8^+^ (**C**) T cell, germinal center B cell (**D**) responses in ILN were assessed by flow cytometry. Data are expressed as mean ± SD (* *p* < 0.05, ** *p* < 0.01, *** *p* < 0.001; ns: no statistical significance).

**Figure A3:**
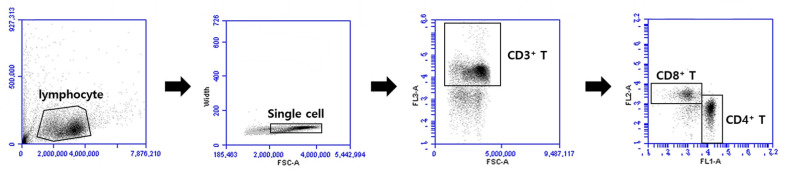
The gating strategy for CD4^+^ and CD8^+^ T cells. Lymphocytes were gated as indicated to analyze the frequencies of CD4^+^ and CD8^+^ T cells by flow cytometry.

At 6 dpi, the blood and ILN samples were collected from mice, and the single cell populations were prepared to analyze the CD4^+^, CD8^+^ T cell, and germinal center B cell frequencies by flow cytometry. A sample gating strategy for flow cytometric analysis of T cells has been provided (Figure A3). Both rVV-AMA1 and rVV-MIC immunization elicited significantly enhanced CD4^+^ T cell frequencies compared to naïve challenge groups (Figure 4A, * *p* < 0.05). However, blood CD8^+^ T cell populations were negligibly changed (Figure 4B). In the ILN, significant differences in CD8^+^ T cell proliferation were observed across the groups. While enhanced CD8^+^ T cell frequencies were detected from both AMA1 and MIC rVV vaccines, induction was significantly greater in the latter of the two (Figure 4C; * *p* < 0.05, ** *p* < 0.01, *** *p* < 0.001). A similar trend was observed for the GC B cells. Recombinant vaccinia virus vaccines significantly elevated the expression of GC B cells, with higher induction occurring in the ILNs of the rVV-MIC immunization group (Figure 4D; * *p* < 0.05, ** *p* < 0.01, *** *p* < 0.001).

### 3.4. Inflammatory Cytokine Production in Splenocytes

**Figure 5:**
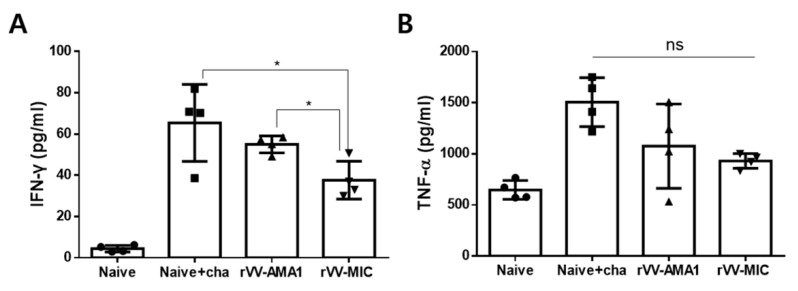
Inflammatory cytokine production in the spleen. Inflammatory cytokine productions were assessed from the spleens of mice at 6 dpi with *P. berghei*. Splenic IFN-γ (**A**) and TNF-α (**B**) cytokines were investigated using the BD OptEIA IFN-γ, TNF-α ELISA kits. Data are expressed as mean ± SD (* *p* < 0.05; ns: no statistical significance).

Individual spleens were collected and homogenized to determine the levels of inflammatory cytokines IFN-γ and TNF-α. Production of the two inflammatory cytokines was assessed using the spleen homogenate supernatant. Significantly reduced splenic IFN-γ levels were observed in the rVV-MIC immunization group. While a marginal reduction in IFN-γ was observed from the rVV-AMA1 immunized mice, the vaccine-induced changes were negligible (Figure 5A, * *p* < 0.05). Contrary to this finding, statistical significance between the groups was not observed for the splenic TNF-α levels (Figure 5B).

### 3.5. Parasitemia, Bodyweight Reduction, and Survival Rate

**Figure 6:**
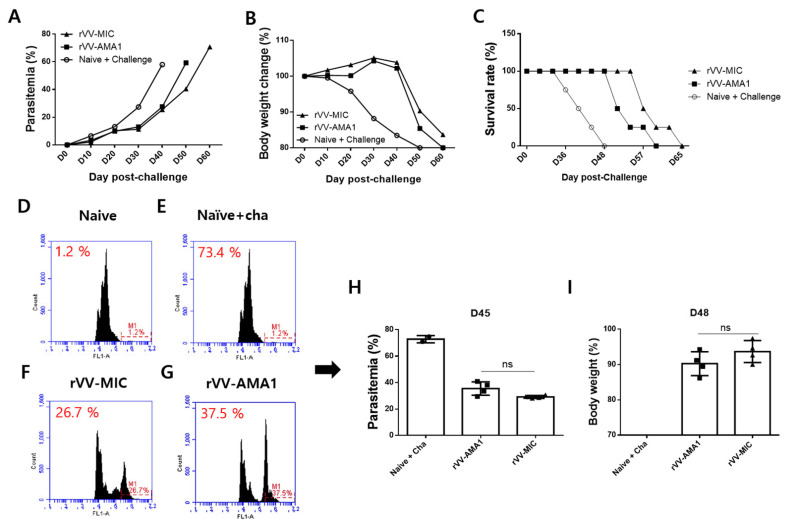
Parasitemia, body weight, and survival rate. After immunization with the rVV-AMA1 or rVV-MIC vaccines, mice (*n* = 8) were infected intraperitoneally with 0.05% of *P. berghei* and monitored at regular intervals to assess changes in parasitemia, body weight, and survival. Parasitemia (**A**), body weight changes (**B**), and survival rate (**C**) from days 0 to 65 post-challenge infections have been monitored. The highest differences in parasitemia (**D**–**H**) and body weight (**I**) between immunized mice and non-immunized naïve control were observed at days 45 and 48 post-challenge infections, respectively. ns: no statistical significance.

As seen in Figure 6, kinetic changes for parasitemia (A), body weight changes (B), and survival rate (C) between immunized mice and non-immunized control (Naïve + challenge) from days 0 to day 65 post-challenge were monitored. On day three post-challenge, 2–3% of parasitemia with 1.2% of this value corresponding to the fluorescence background as demonstrated from naïve mice (D-H). On day 45 post-challenge, parasitemia in the naïve + cha, rVV-MIC, and rVV-AMA1 were 73.4%, 26.7%, and 37.5% each, respectively (Figure 6D–H). Interestingly, lower levels of parasitemia were observed from rVV-MIC compared to rVV-AMA1 immunization. Compared to the naïve + cha group, rVV-AMA1 and rVV-MIC immunization prolonged the survival of mice by 12 and 17 days, respectively (Figure 6C). The highest differences in parasitemia (H) and body weight (I) between immunized mice and non-immunized naïve control were observed at days 45 and 48 post-challenge infections, respectively, thus indicating that protections were induced in both AMA1 and MIC groups.

## 4. Discussion

The complex life cycle of *Plasmodium* spp. continues to hinder the development of an efficacious malaria vaccine; a monumental task that remains unresolved. Currently, many of the blood-stage vaccines are designed based on the surface antigens of *Plasmodium* spp., such as the AMA1. A major drawback of the AMA1 antigen is its highly polymorphic nature, resulting in allele-specific immune protection [8,20]. Consequently, AMA1-based vaccines failed to demonstrate protective efficacies in controlled clinical trials [21]. To address these recurring issues with the antigens, we explored the potential of MIC antigen as a vaccine candidate. Our findings revealed that rVV-MIC immunization elicited stronger antibody responses after 2nd boost immunization. Although CD4^+^ and CD8^+^ T cell responses from the blood were comparable between rVV-AMA1 and rVV-MIC immunized mice, ILN CD8^+^ T cell and GC B cell inductions were significantly greater for the rVV-MIC. This fact, paired with reduced splenic inflammatory cytokine production, contributed to enhanced survival and lessened parasitemia in the blood of rVV-MIC immunized mice.

The majority of the findings presented here are consistent with the literature, especially those of our previous studies. In our previous study, immunization of *P. berghei* MSP-8 and MSP-9 virus-like particles (VLPs) significantly lessened pro-inflammatory cytokine IFN-γ [22,23]. Consistent with these previous results, the rVV vaccines used in our study elicited a significant reduction of the inflammatory cytokine IFN-γ while changes to TNF-α were negligible. Germinal center B cells are an integral component of the humoral immune response, with their roles in memory B cell and long-lived plasma cell production [24]. In our study, antigen-specific GC B cells in inguinal lymph nodes were higher in the immunized group, contributing to increasingly high levels of parasite-specific IgG antibody response. These effects combined led to reduced parasitemia, marginal bodyweight change, and prolonged survival of immunized mice. Differences in TNF-α were also observed compared to results from others that assessed the efficacies of vaccines against *P. berghei* ANKA. Immunizing mice with the macrophage migration inhibitory factor ortholog produced in *Plasmodium* spp. reduced the production of TNF-α cytokines [25]. Similar to this finding, while the vaccinia virus-vectored vaccines used in the present study reduced the production of TNF-α, the changes were not statistically significant.

There were some striking differences in survival compared to our previous studies, but the underlying cause of this discrepancy can be attributed to the challenge infection dose and immunization schedule. In both of our previous studies, mice were challenge-infected with 1 × 10^5^ or 1 × 10^4^ *P. berghei* via IP routes following a prime-boost regimen [15,22]. Here, we used 5 × 10^4^ *P. berghei* for challenge infection dose with three vaccine immunizations for mice, which may have led to longer survival duration for both control and immunized mice. GC B cell responses from the MVA-based vaccines were much weaker than the findings presented in our VLP vaccine study [15]. A possible explanation for this phenomenon could be the vaccine platform used in the study. One study reported that MVA-induced antigen-specific GC B cell and antibody responses were much weaker than adenovirus vectored vaccines or the protein subunit vaccines [26]. Another possibility is due to the parasitic nature of *Plasmodium* spp, particularly during the blood stage of its life cycle. It has been reported that the blood stage infection by *Plasmodium* spp. can interfere with the differentiation of CSP-specific GC B cells, consequently leading to aberrant humoral response [27]. Given that high levels of type I inflammatory cytokines such as IFN-γ can inhibit GC B cell response in *P. berghei* ANKA infection, this is another possibility [28].

RTS, S vaccine is the most advanced malaria vaccine to date, and its development served as a giant step forward for malaria vaccines. Unfortunately, the clinical endpoint results indicated that this vaccine failed to confer durable protection [2]. Several factors are thought to be associated with the low efficacy of the RTS,S vaccine, including the intensity of malaria transmission in the region and the reduced levels of circulating antibodies in vaccinees [29]. In our study, high levels of parasite-specific antibodies were detected. Yet, the durability of these antibody responses and the extent of their contribution to protection against rodent malaria requires further elucidation prior to evaluation using human malaria. Similar to the RTS, S vaccine, the protection conferred by the vaccine used in the present study was incomplete as all of the immunized mice eventually succumbed to death, thus signifying the need for additional improvements.

It is noteworthy to mention that genomic databases are not well-characterized for *P. berghei*. Evidently, the MIC protein used in our experiment was a putative protein with an unknown function. Nevertheless, immunization with the *P. berghei* MIC resulted in protection exceeding that of the rVV-AMA1 vaccine. For this reason, an actual immunoproteomic study elucidating the function of the protein used in this study must be performed for further development. Notably, the microneme organelle contains numerous proteins involved in various parasitic functions and surprisingly, many of these proteins were reported to be conserved between *P. falciparum* and *P. berghei* [30]. Surprisingly, some multiple microneme-derived proteins, such as the AMA1, were also highly conserved across the phylum Apicomplexa. These interesting findings suggest microneme antigen-based vaccines targeting other apicomplexan parasites could also be employed as an antimalarial vaccine. For instance, multiple microneme proteins are highly conserved and functionally interchangeable between *T. gondii* and *Plasmodium* spp., despite the large differences in their sequence identities [31,32]. Several microneme antigen-based experimental vaccines have been reported for *T. gondii*. DNA vaccines constructed using the *T. gondii* microneme antigen fragments conferred protection in mice, indicated by an 84% brain cyst burden reduction [33]. In our previous study, VLP vaccines expressing the *T. gondii* microneme protein eight protected mice against the virulent *T. gondii* RH strain [17]. Based on these reports, applying the *T. gondii* MIC-based vaccines to target malaria could prove to be useful and investigating the feasibility of this vaccination approach would be interesting.

## 5. Conclusions

The rVV vaccines expressing the *P. berghei* AMA1 or MIC antigens induced humoral and cellular immune responses, which aided in lessening pro-inflammatory cytokine production and parasitemia of immunized mice to prolong their survival. Further studies investigating methods to enhance their efficacies, such as incorporating heterologous immunization strategies, should be conducted to develop successful antimalarial blood-stage vaccines.

## Data Availability

The original contributions presented in this study are included in the article. Further inquiries can be directed to the corresponding author.

## References

[1] WHO (2021). World Malaria Report 2021.

[2] Olotu A., Fegan G., Wambua J., Nyangweso G., Leach A., Lievens M., Kaslow D.C., Njuguna P., Marsh K., Bejon P. (2016). Seven-Year Efficacy of RTS,S/AS01 Malaria Vaccine among Young African Children. N. Engl. J. Med..

[3] Price P.J., Torres-Domínguez L.E., Brandmüller C., Sutter G., Lehmann M.H. (2013). Modified Vaccinia virus Ankara: Innate immune activation and induction of cellular signalling. Vaccine.

[4] Gilbert S.C. (2013). Clinical development of Modified Vaccinia virus Ankara vaccines. Vaccine.

[5] de Barra E., Hodgson S.H., Ewer K.J., Bliss C.M., Hennigan K., Collins A., Berrie E., Lawrie A.M., Gilbert S.C., Nicosia A. (2014). A phase Ia study to assess the safety and immunogenicity of new malaria vaccine candidates ChAd63 CS administered alone and with MVA CS. PLoS ONE.

[6] Sheehy S.H., Duncan C.J., Elias S.C., Biswas S., Collins K.A., O’Hara G.A., Halstead F.D., Ewer K.J., Mahungu T., Spencer A.J. (2012). Phase Ia clinical evaluation of the safety and immunogenicity of the Plasmodium falciparum blood-stage antigen AMA1 in ChAd63 and MVA vaccine vectors. PLoS ONE.

[7] Biswas S., Choudhary P., Elias S.C., Miura K., Milne K.H., de Cassan S.C., Collins K.A., Halstead F.D., Bliss C.M., Ewer K.J. (2014). Assessment of humoral immune responses to blood-stage malaria antigens following ChAd63-MVA immunization, controlled human malaria infection and natural exposure. PLoS ONE.

[8] Takala S.L., Coulibaly D., Thera M.A., Batchelor A.H., Cummings M.P., Escalante A.A., Ouattara A., Traoré K., Niangaly A., Djimdé A.A. (2009). Extreme polymorphism in a vaccine antigen and risk of clinical malaria: Implications for vaccine development. Sci. Transl. Med..

[9] Yan M., Cui X., Zhao Q., Zhu S., Huang B., Wang L., Zhao H., Liu G., Li Z., Han H. (2018). Molecular characterization and protective efficacy of the microneme 2 protein from Eimeria tenella. Parasite.

[10] Hernández-Silva D.J., Valdez-Espinoza U.M., Mercado-Uriostegui M.A., Aguilar-Tipacamú G., Ramos-Aragón J.A., Hernández-Ortiz R., Ueti M., Mosqueda J. (2018). Immunomolecular Characterization of MIC-1, a Novel Antigen in Babesia bigemina, Which Contains Conserved and Immunodominant B-Cell Epitopes that Induce Neutralizing Antibodies. Vet. Sci..

[11] Monney T., Rütti D., Schorer M., Debache K., Grandgirard D., Leib S.L., Hemphill A. (2011). RecNcMIC3-1-R is a microneme- and rhoptry-based chimeric antigen that protects against acute neosporosis and limits cerebral parasite load in the mouse model for Neospora caninum infection. Vaccine.

[12] Arumugam T.U., Takeo S., Yamasaki T., Thonkukiatkul A., Miura K., Otsuki H., Zhou H., Long C.A., Sattabongkot J., Thompson J. (2011). Discovery of GAMA, a Plasmodium falciparum merozoite micronemal protein, as a novel blood-stage vaccine candidate antigen. Infect. Immun..

[13] Changrob S., Han J.H., Ha K.S., Park W.S., Hong S.H., Chootong P., Han E.T. (2017). Immunogenicity of glycosylphosphatidylinositol-anchored micronemal antigen in natural Plasmodium vivax exposure. Malar. J..

[14] De Niz M., Heussler V.T. (2018). Rodent malaria models: Insights into human disease and parasite biology. Curr. Opin. Microbiol..

[15] Lee D.H., Chu K.B., Kang H.J., Lee S.H., Chopra M., Choi H.J., Moon E.K., Inn K.S., Quan F.S. (2019). Protection induced by malaria virus-like particles containing codon-optimized AMA-1 of Plasmodium berghei. Malar. J..

[16] Cao Y., Zhang D., Pan W. (2009). Construction of transgenic Plasmodium berghei as a model for evaluation of blood-stage vaccine candidate of Plasmodium falciparum chimeric protein 2.9. PLoS ONE.

[17] Lee S.H., Kim A.R., Lee D.H., Rubino I., Choi H.J., Quan F.S. (2017). Protection induced by virus-like particles containing Toxoplasma gondii microneme protein 8 against highly virulent RH strain of Toxoplasma gondii infection. PLoS ONE.

[18] Lim J.F., Berger H., Su I.H. (2016). Isolation and Activation of Murine Lymphocytes. J. Vis. Exp. JoVE.

[19] Somsak V., Srichairatanakool S., Yuthavong Y., Kamchonwongpaisan S., Uthaipibull C. (2012). Flow cytometric enumeration of Plasmodium berghei-infected red blood cells stained with SYBR Green I. Acta Trop..

[20] Duan J., Mu J., Thera M.A., Joy D., Kosakovsky Pond S.L., Diemert D., Long C., Zhou H., Miura K., Ouattara A. (2008). Population structure of the genes encoding the polymorphic Plasmodium falciparum apical membrane antigen 1: Implications for vaccine design. Proc. Natl. Acad. Sci. USA.

[21] Payne R.O., Milne K.H., Elias S.C., Edwards N.J., Douglas A.D., Brown R.E., Silk S.E., Biswas S., Miura K., Roberts R. (2016). Demonstration of the Blood-Stage Plasmodium falciparum Controlled Human Malaria Infection Model to Assess Efficacy of the P. falciparum Apical Membrane Antigen 1 Vaccine, FMP2.1/AS01. J. Infect. Dis..

[22] Lee S.H., Kang H.J., Chu K.B., Basak S., Lee D.H., Moon E.K., Quan F.S. (2020). Protective Immunity Induced by Virus-Like Particle Containing Merozoite Surface Protein 9 of Plasmodium berghei. Vaccines.

[23] Lee S.H., Chu K.B., Kang H.J., Basak S., Kim M.J., Park H., Jin H., Moon E.K., Quan F.S. (2020). Virus-like particles expressing Plasmodium berghei MSP-8 induce protection against P. berghei infection. Parasite Immunol..

[24] Shlomchik M.J., Weisel F. (2012). Germinal center selection and the development of memory B and plasma cells. Immunol. Rev..

[25] Baeza Garcia A., Siu E., Sun T., Exler V., Brito L., Hekele A., Otten G., Augustijn K., Janse C.J., Ulmer J.B. (2018). Neutralization of the Plasmodium-encoded MIF ortholog confers protective immunity against malaria infection. Nat. Commun..

[26] Wang C., Hart M., Chui C., Ajuogu A., Brian I.J., de Cassan S.C., Borrow P., Draper S.J., Douglas A.D. (2016). Germinal Center B Cell and T Follicular Helper Cell Responses to Viral Vector and Protein-in-Adjuvant Vaccines. J. Immunol..

[27] Keitany G.J., Kim K.S., Krishnamurty A.T., Hondowicz B.D., Hahn W.O., Dambrauskas N., Sather D.N., Vaughan A.M., Kappe S.H.I., Pepper M. (2016). Blood Stage Malaria Disrupts Humoral Immunity to the Pre-erythrocytic Stage Circumsporozoite Protein. Cell. Rep..

[28] Ryg-Cornejo V., Ioannidis L.J., Ly A., Chiu C.Y., Tellier J., Hill D.L., Preston S.P., Pellegrini M., Yu D., Nutt S.L. (2016). Severe Malaria Infections Impair Germinal Center Responses by Inhibiting T Follicular Helper Cell Differentiation. Cell. Rep..

[29] White M.T., Verity R., Griffin J.T., Asante K.P., Owusu-Agyei S., Greenwood B., Drakeley C., Gesase S., Lusingu J., Ansong D. (2015). Immunogenicity of the RTS,S/AS01 malaria vaccine and implications for duration of vaccine efficacy: Secondary analysis of data from a phase 3 randomised controlled trial. Lancet Infect. Dis..

[30] Dubois D.J., Soldati-Favre D. (2019). Biogenesis and secretion of micronemes in Toxoplasma gondii. Cell. Microbiol..

[31] Carruthers V.B., Tomley F.M., Burleigh B.A., Soldati-Favre D. (2008). Microneme Proteins in Apicomplexans. Molecular Mechanisms of Parasite Invasion: Subcellular Biochemistry.

[32] Kappe S., Bruderer T., Gantt S., Fujioka H., Nussenzweig V., Ménard R. (1999). Conservation of a gliding motility and cell invasion machinery in Apicomplexan parasites. J. Cell. Biol..

[33] Beghetto E., Nielsen H.V., Del Porto P., Buffolano W., Guglietta S., Felici F., Petersen E., Gargano N. (2005). A combination of antigenic regions of Toxoplasma gondii microneme proteins induces protective immunity against oral infection with parasite cysts. J. Infect. Dis..

